# Changing profile of rotavirus genotypes in Bangladesh, 2006–2012

**DOI:** 10.1186/1471-2334-13-320

**Published:** 2013-07-15

**Authors:** Mokibul Hassan Afrad, Zahid Hassan, Saiada Farjana, Sayra Moni, Subarna Barua, Sumon Kumar Das, Abu Syed Golam Faruque, Tasnim Azim, Mustafizur Rahman

**Affiliations:** 1 Virology Laboratory, International Centre for Diarrhoeal Disease Research, Bangladesh (icddr,b), Mohakhali, Dhaka 1212, Bangladesh

**Keywords:** Rotavirus, Diarrhea, Vaccine, Bangladesh, Genotype, Epitope

## Abstract

**Background:**

Rotavirus is the leading cause of severe diarrhea in infants and young children worldwide including Bangladesh. Unlike what was seen in high-income countries, the licensed rotavirus vaccines did not show high efficacy in Bangladeshi trials. We assessed rotavirus prevalence and genotypes in Bangladesh over six-year period to provide baseline information on the rotavirus burden and changing profile in the country.

**Methods:**

This study was conducted from June 2006 to May 2012 in Matlab, Bangladesh. Group A rotaviruses were detected in stools collected from diarrhea patients by ELISA and genotyped using multiplex reverse transcription PCR followed by nucleotide sequencing.

**Results:**

Of the 9678 stool samples, 20.3% were positive for rotavirus. The most predominant genotype was G1P[8] (22.4%), followed by G9P[8] (20.8%), G2P[4] (16.9%) and G12P[8] (10.4%). Mixed infections were detected in 14.2% of the samples. Emergence of an unusual strain, G9P[4] was documented during 2011–12. Several amino acid mismatches in the antigenic epitopes of VP7 and VP4 between Bangladeshi and the vaccine strains were identified.

**Conclusions:**

Our study provides important information on rotavirus genotypes that should be considered for the selection and introduction of rotavirus vaccines in Bangladesh.

## Background

Rotavirus causes severe diarrhea in infants and young children worldwide and is responsible for an estimated 475,000-580,000 deaths and >2 million hospitalizations each year [1]. Most of these deaths occur in developing countries especially in Africa and Asia [2]. The virus is commonly characterized by the presence of two neutralizing antigens on their outer capsid proteins, VP7 (G genotypes) and VP4 (P genotypes) [3]. To date, at least 27 G and 37 P genotypes have been described in humans and animal species [4,5]. Until the mid-1990s, the most common human rotavirus types were G1P[8], G2P[4], G3P[8], and G4P[8]. Two additional types G9 and G12 associated with P[8] or P[6] have emerged since 1995 and 2001, respectively, and have become common in humans [6,7].

The current rotavirus vaccines are based on the strains that were isolated in the 1980s. Rotarix™ is derived from the attenuated human G1P[8] rotavirus group A strain 89–12 [8], while RotaTeq™ contains five human-bovine reassortant rotavirus group A strains, WI79-9 (G1), SC2-9 (G2), WI78-9 (G3), BrB-9 (G4), and WI79-4 (P[8]). These vaccines have demonstrated high efficacy (>90%) against severe rotavirus disease in high and middle income countries [9-12]. However, the clinical trials failed to confer adequate efficacy (<60%) in low income countries such as India, Malawi, Nicaragua, Vietnam, and Bangladesh [13-16]. A key question that remains is why rotavirus vaccines did not provide sufficient protection in low income countries. One of the reasons could be the strain diversity and antigenic variations of rotavirus strains compared to the vaccine strains. Lower vaccine efficacy may also be caused by the presence of sub-genotypic lineages. Hoshino *et al*. showed that antisera raised against G9 lineage 1 strains had a broad neutralizing ability against all three G9 lineages, while antisera raised against G9 lineage 2 or 3 strains had a lower neutralizing ability to other G9 lineages [17]. Moreover, accumulation of point mutations in the antigenic epitopes could drive the virus to escape the vaccine induced immunity. Prior to 1995, 96.3% of all reported rotavirus strains matched antigens present in either RotaTeq™ or Rotarix™ vaccines (G1-G4). However, the proportion of vaccine-matched strains declined to 70.5% by 2005–2009. The VP7 trimer, which is responsible for evoking neutralizing antibodies, contains two structurally defined antigenic epitopes: 7–1 and 7–2. The 7–1 epitope spans the inter-subunit boundary and is further subdivided into 7-1a and 7-1b. Activation of another neutralizing protein VP4 requires its proteolytic cleavage into VP8 and VP5 which contain four (8–1 to 8–4) and five (5–1 to 5–5) surface-exposed antigenic epitopes respectively [18].

Since 1963, International Centre for Diarrhoeal Disease Research, Bangladesh (icddr,b) has maintained a treatment facility in rural Matlab that provides healthcare services to 12,000-15,000 diarrhea patients annually. It has also established a surveillance system to monitor the common diarrhea pathogens that have shown rotavirus as the predominant diarrheal etiological agent. RotaTeq™ and Rotarix™ vaccine trials conducted in Matlab during 2007–2012 showed vaccine efficacy ofless than 45% [13], which is much lower than what has been observed in high income countries. To better understand whether the lower immune response was related to differences in circulating versus vaccine strains, this study assessed the rotavirus prevalence and genotypic variability in Matlab. Such information is pertinent for making decisions for selection of suitable vaccine strains for Bangladesh.

## Methods

### Study population

The study was conducted in rural Matlab, Bangladesh during June 2006- May 2012. Matlab is a low-lying riverine area located 55 km southeast of Dhaka and has a population of approximately 300,000. Stool specimens were obtained from an ongoing rotavirus surveillance ‘The Diarrhoeal Disease Surveillance System (DDSS)’, which has been approved by the Research Review Committee (RRC) and Ethical Review Committee (ERC) of icddr,b in accordance with the Helsinki Declaration on ethical principles for medical research involving human subjects. Informed written consent as well as accent has been taken from the care givers or guardians on behalf of the patients. All the specimens were properly labeled with information, including a unique identification number and the date of collection. The specimens were stored temporarily in refrigerators at 4-8°C prior to transport to the icddr,b Virology Laboratory in Dhaka in cold-boxes with ice-packs.

### Detection of rotavirus

Rotavirus antigen (group A rotavirus-specific VP6 proteins) was detected in the stool specimens using a solid-phase sandwich-type enzyme immunoassay (EIA) modeled after the Dakopatts commercial kit (Dakopatts, Copenhagen, Denmark), incorporating rabbit hyperimmune antisera produced at icddr,b and an anti-human rotavirus-horseradish peroxidase conjugate [19].

### Genotyping

Statistically, at 95% confidence level with a margin error of 7%, the desired total sample size for genotyping was calculated to be approximately 183, 126, 151 and 12 for genotype G1P[8], G2P[4], G9P[8] and G12P[8], respectively in regards to the previous published genotype prevalence data in Matlab [19]. However, the number of samples genotyped in each year was low and this is the limitation of this study. We cannot rule out that the low numbers of samples might have influenced the yearly distribution of rotavirus genotypes and that a larger number of samples could have altered the relative proportion of the rotavirus strains.

In this study, every tenth, n=183 (10% of the total samples) rotavirus EIA positive stool samples were systematically enrolled for genotyping irrespective of age, sex, socio-demographic and nutritional status. Genomic RNA was extracted using the QIAamp Viral RNA mini kit (Qiagen/Westburg, Leusden, the Netherlands) according to the manufacturer’s instructions. Multiplex reverse transcription polymerase chain reaction (RT-PCR) was performed using Qiagen One-Step RT-PCR Kit (Qiagen, Hilden, Germany) as previously described [19]. The extracted RNA was denatured at 97°C for 3 min. Briefly, the reaction was carried out with an initial reverse transcription step at 45°C for 30 min, followed by 35 cycles of amplification (30s at 94°C, 30s at 48°C, 60s at 72°C), and a final extension of 7 min at 72°C in a thermal cycler (Eppendorf AG, Hamburg, Germany). PCR products were run on a 1.5% agarose gel, stained with ethidium bromide and visualized under UV-light.

### Polyacrylamide gel electrophoresis (PAGE)

The standard phenol-chloroform extraction and alcohol precipitation methods were used for the extraction of viral RNA from stool samples using aphenol:chloroform:isoamylalcohol (25:24:1) mixture as described elsewhere [20]. The RNA was separated by polyacrylamide gel electrophoresis for 18 hours at 100 volts [20,21]. The RNA migration pattern of 11 segments of dsRNA was identified by staining the gel with silver nitrate.

### Nucleotide sequencing

Samples that were untypeable by multiplex PCR were characterized by sequencing with primers Beg9/End9 (VP7) and VP4-117F/Con2 (VP4). In brief, RT-PCR was performed and products were purified using ExoSAP-IT (USB Corp, Cambridge, MA). Cycle-sequencing reactions were carried out using the dideoxynucleotide chain termination method with the ABI PRISM® BigDye Terminator Cycle Sequencing Reaction kit v3.1 (Perkin-Elmer Applied Biosystems, Foster City, CA) in an automated genetic analyzer (ABI 3500xL). The chromatogram sequencing files were inspected using Chromas 2.3 (Technelysium, Helensvale, Australia). Sequences were compared with existing rotavirus sequences in the NR/NT database using the BLASTN program at the National Center for Biotechnology Information website (available at: http://blast.ncbi.nlm.nih.gov).

For antigenic characterization, sequences were aligned in Sequencher 5.0 (Gene Codes Corporation, Ann Arbor, MI). The sequences obtained from the study were submitted to GenBank under the accession numbers KC484719 through KC484726.

## Results

### Detection of rotavirus

**Figure 1:**
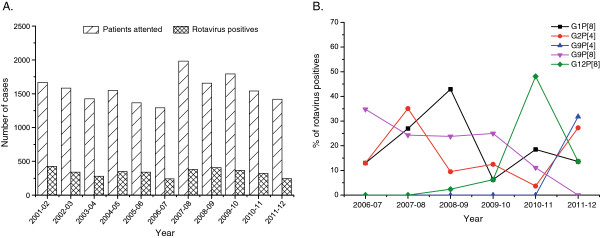
**Prevalence and genotype distribution of rotaviruses in Matlab, Bangladesh. **A: Number of diarrhea patients attended and number of cases tested rotavirus positive, June 2001-May 2012. B: Temporal changes in the distribution of major rotavirus genotypes, June 2006-May 2012.

From June 2006 to May 2012, a total of 9678 patients with acute diarrhea attending Matlab hospital, of which 1963 (20.3%) were rotavirus positive. Figure 1A shows the yearly distribution of rotavirus hospitalization during our study period together with the previously published data of Matlab from June 2001 to May 2006. We found that rotavirus was represented in a remarkable proportion of diarrhea hospitalizations with the highest detection rate (24.5%) in 2008–09 and the lowest (17.3%) in 2011–12 (Table 1).

**Table 1:** Distribution of specimens positive for rotavirus, Bangladesh, June 2006-May 2012

	**2006-07**	**2007-08**	**2008-09**	**2009-10**	**2010-11**	**2011-12**	**Total**
**Rotavirus detection: number (%)**							
Samples tested	1291	1982	1656	1791	1539	1419	9678
Rotavirus positive	240 (18.6)	382 (19.3)	406 (24.5)	368 (20.5)	321 (20.9)	246 (17.3)	1963 (20.3)
**Rotavirus genotype distribution (10% Rotavirus positives): number (%)**							
G1P[8]	3 (13)	10 (27)	18 (42.9)	2 (6.3)	5 (18.5)	3 (13.6)	41 (22.4)
G2P[4]	3 (13)	13 (35.1)	4 (9.5)	4 (12.5)	1 (3.7)	6 (27.3)	31 (16.9)
G9P[8]	8 (34.8)	9 (24.3)	10 (23.8)	8 (25)	3 (11.1)	0	38 (20.8)
G12P[8]	0	0	1 (2.4)	2 (6.3)	13 (48.1)	3 (13.6)	19 (10.4)
**Subtotal**	**14 (60.9)**	**32 (86.5)**	**33 (78.6)**	**16 (50.0)**	**22 (81.5)**	**12 (54.5)**	**129 (70.5)**
G1P[6]	1 (4.3)	0	2 (4.8)	0	0	0	3 (1.6)
G2P[6]	0	1 (2.7)	0	0	0	0	1 (0.5)
G2P[8]	0	1 (2.7)	1 (2.4)	0	0	0	2 (1.1)
G4P[8]	1 (4.3)	0	0	0	0	0	1 (0.5)
G9P[4]	0	0	0	0	0	7 (31.8)	7 (3.8)
G9P[6]	2 (8.7)	0	1 (2.4)	1 (3.1)	0	0	4 (2.2)
G12P[4]	0	0	0	0	0	1 (4.5)	1 (0.5)
G12P[6]	0	1(2.7)	3 (7.1)	3 (9.4)	1 (3.7)	0	8 (4.4)
**Subtotal**	**4 (17.4)**	**3 (8.1)**	**7 (16.7)**	**4 (12.5)**	**1 (3.7)**	**8 (36.4)**	**27 (14.8)**
G/P Untypeable	0	0	0	0	0	1 (4.5)	1 (0.5)
Mixed G/P^*^	5 (21.7)	2 (5.4)	2 (4.8)	12 (37.5)	4 (14.8)	1 (4.5)	26 (14.2)
**Total typed**	**23 (100)**	**37 (100)**	**42 (100.1)**	**32 (100)**	**27 (100)**	**22 (100)**	**183 (100)**

^*^Mixed G and P types: 2006–07: G1G9P[8] = 3, G1G4P[6] = 1, G2G9P[8] = 1; 2007–08: G2G9P[8] = 1, G1G2G9P[8] = 1; 2008–09: G1G9P[8] = 2; 2009–10: G1G2P[4] = 2, G2G9P[4] = 1, G2G9P[8] = 2,G1G2G9P[8] = 1, G1G2G9P[4]P[6]P[8] = 1, G1G2G9P[6]P[8] = 2, G1G2G9P[6] = 1, G2G9P[4]P[8] = 1, G1P[6]P[8] = 1; 2010–11: G1G12P[8] = 4; 2011–12: G2G9G12P[8] = 1

### Distribution of G and P genotypes

We performed G and P genotyping of 183 rotavirus strains and found that five G genotypes (G1, G2, G4, G9 and G12) combined mostly with three P genotypes (P[8], P[4] and P[6]) were circulating (Table 1). Among them, globally common circulating strains G1P[8], G2P[4], G9P[8] and G12P[8] accounted for 70.5% of the total samples. Overall, G1P[8] (22.4%) was most prevalent followed by G9P[8] (20.8%), G2P[4] (16.9%) and G12P[8] (10.4%). We identified 14.8% uncommon strains including G1P[6], G2 with P[6] and P[8], G4P[8], G9 with P[4] and P[6] and G12 with P[4] and P[6] throughout the study. We detected a substantial proportion of mixed infections (14.2%) that were further confirmed by PAGE. The highest incidence of mixed infections was observed in 2009–10 (37.5%). Among them, P[8] associated with G1, G2, G9 and G12 accounted for the majority.

### Fluctuation of the G and P types

We observed a wide fluctuation of rotavirus genotypes during our study period. Figure 1B shows the overall distribution of the major genotypes from June 2006 to May 2012. Genotype G1P[8] was the predominant (42.9%) in 2008–09, sharply decreased in 2009–10 (6.3%) and this fluctuation continued in the following seasons. G9P[8] genotype was the most common (34.8%) in 2006–07 and declined gradually. G2P[4], which was less common until 2004–05, was prominent in 2005–06, 2007–08, and 2011–12. G12P[8], which was detected infrequently before 2009–10, sharply increased in 2010–11 (48.1%).

### Antigenic relationship with vaccine strains

We compared the antigenic properties of the most recent rotavirus strains circulating in Matlab with the neutralizing epitopes of the VP7 and VP4 proteins of the vaccine strains (RotaTeq™ and Rotarix™). Phylogenetic analysis (data not shown) revealed that all Bangladeshi G1 genotypes clustered in lineage 1, G2 in lineage 4, G9 and G12 in lineage 3, P[4] in lineage 1 and P[8] in lineage 3 with >99% nucleotide identity intra-genotypically. Therefore, we included one representative strain from each genotype (G1, G2, G9, G12, P[8] and P[4]) in the final analysis.

**Figure 2:**
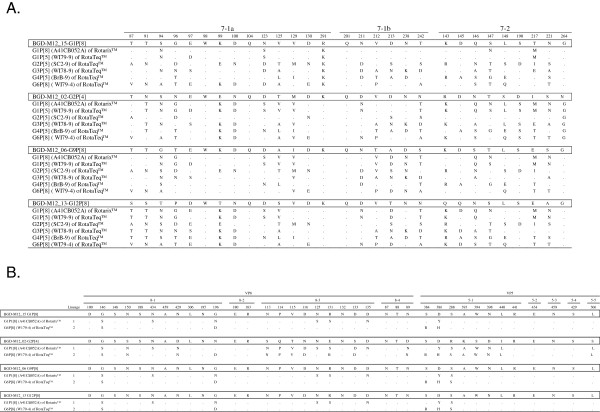
**Amino acid substitutions among Bangladeshi rotavirus strains (G1, G2, G9, G12 and P[8]) and vaccine strains in RotaTeq™ (G1, G2, G3, G4 and P**[8]**) and Rotarix™ (G1 and P**[8]**).** Differences in amino acids were calculated from the epitopic regions of the VP7 and VP4 proteins. BGD, Bangladesh.

We compared the amino acid residues in antigenic epitopes of VP7 and VP4 between vaccine strains and Bangladeshi strains (Figure 2). Of the 29 amino acid residues of VP7 epitopes situated in 7-1a, 7-1b and 72 antigenic sites, only three (position 98, 104, and 201) were completely conserved among all Bangladeshi and vaccine strains. The Bangladeshi G1 strain showed 4 differences (positions at 94, 123, 217, and 291) with the G1 strains of both RotaTeq™ and Rotarix™ and three of them were located at 7-1a and one in 7–2 epitope. In addition, one amino acid at position 97 was different in RotaTeq™ when compared to Rotarix™ and Bangladeshi G1 strain. As expected, the differences between Bangladeshi G1 strain and other strains in RotaTeq™ were much higher (> 14 amino acid). The Bangladeshi G2 strain showed 6 amino acid differences with RotaTeq™ G2 strain, most of which were located in 7-1a and 7-1b. On the other hand, heterotypes in both vaccines showed that at least 18 amino acid differences were present. Comparative analysis of Bangladeshi G9 and G12 strains thathave not been included in the vaccines revealed that the number of amino acid differences were much higher (up to 21 for G12 and 18 for G9). However, G9 was more closely related to the vaccines than G12 based on antigenic sites. The VP4 antigenic epitopes of the Bangladeshi P[8] strains were very close to both vaccines regardless of G genotypes (maximum 5 amino acid differences out of 37 with RotaTeq™ and 8 with Rotarix™). Expectedly, Bangladeshi P[4] strain showed the greatest divergence from the P[8] epitopes of the vaccines having 20 amino acid changes with Rotarix™ and 19 with RotaTeq™. Most of these changes were located at 8–3 and 8–4 epitopes.

## Discussion

From 2007 to 2012, icddr,b conducted two rotavirus vaccine trials (RotaTeq™ and Rotarix™) in Matlab. The Government of Bangladesh has decided to include the vaccines in the National Health Immunization Program in the upcoming years. In this study, we aimed to extend the previous rotavirus genotype surveillance conducted in Matlab (2001–06) [19] up to the year 2012 to provide the baseline information for the policy makers to implement the appropriate vaccines in the country.

During our study period, we detected rotavirus throughout the year and did not find a noticeable difference in rotavirus hospitalization compared to aprevious study (Figure 1A). The incidence rate is comparable to the reports from India, Nepal, Malaysia, Japan and Taiwan (20-25%) but much lower than Thailand, Myanmar and Vietnam (38.1-56%) [22]. We observed strong yearly fluctuations (Figure 1B) of genotypes G1P[8], G2P[4], G9P[8] and G12P[8], which accounted for 70.5% of the total strains. In 2010–11, we noticed a sudden change of rotavirus genotypes when a sharp decrease of G2P[4] and G9P[8] was accompanied by the emergence of G12P[8] (Figure 1B). In the same year more than one third of the samples exhibited mixed rotavirus strains with G1, G2 and G9. Mixed infections play an important role in generating reassortant unusual strains thatwere evident in many countries including Bangladesh [23,24]. Consequently, we found high numbers (36.4%) of unusual strains in 2011–12 that might be the results of mixed infections existing in the previous year. Interestingly, for the first time in 2011–12, we identified an unusual strain G9P[4] which was the most predominant strain in that year. It is speculated that G9P[4] has evolved from co-infections with G2P[4] and G9P[8]. Similarly, other unusual strains such as G1P[6], G2P[6], G2P[8] and G9P[6] might be generated through reassortment events between commonly circulating rotavirus strains which are supported by the previous reports [25].

With concordance to the previous studies, G3 has been completely absent since 2001 in Bangladesh [19]. In the same way, G4 strain, which was the most common (47%) from 1992 through 1997 in Bangladesh, decreased gradually over time and we did not identify any G4 following 2006–07. Both of these strains showed similar declining patterns in other Southeast Asian countries [23]; however, they still have been frequently detected in other parts of the world [26,27].

**Figure 3:**
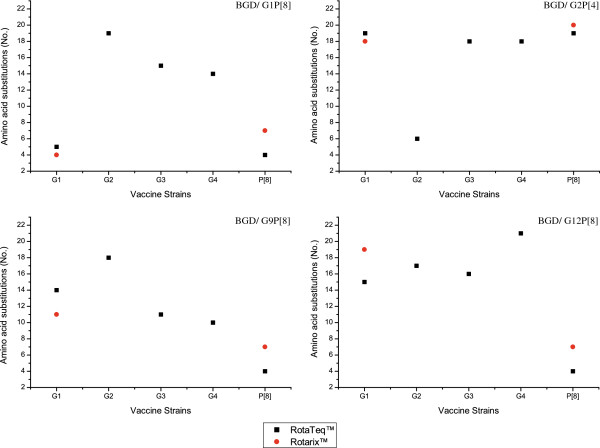
**Amino acid substitutions among Bangladeshi rotavirus strains (G1, G2, G9, G12 and P**[8]**) and vaccine strains in RotaTeq™ (G1, G2, G3, G4 and P**[8]**) and Rotarix™ (G1 and P**[8]**).** Differences in amino acids were calculated from the epitopic regions of the VP7 and VP4 proteins. BGD, Bangladesh.

We found some differences in the antigenic epitopes of the currently circulating homotypic G1, G2 and P[8] genotypes with the vaccine strains (Figure 3). This variation could be due to the fact that the original vaccine strains were isolated quite a long time ago and the circulating strains in the meantime have changed through their natural evolution. Jin and colleagues showed that the propensity of continuing accumulation of point mutations in the antigenic sites of the outer capsid proteins vastly increased the possibility of escaping host immunity conferred by the vaccine virus [28]. As expected, the heterotypic G9, G12 and P[4] genotypes were more distantly related to the vaccine strains compared to the homotypic genotypes. However, genetic variability may not be the concern behind the lower vaccine efficacy in developing countries like Bangladesh due to the fact that most of the concomitantly circulating rotavirus strains in developed and developing countries show a high conservation in antigenic sites. Although our data do not provide a clear indication that the licensed vaccines may be less effective against heterotypes, the apparent emergence of G2P[4] and G9P[4] in the vaccinated areas of Brazil, Mexico, and Australia raises the question whether the shifts of rotavirus strains were the result of natural yearly fluctuation of genotypes or due to the selection pressure [24-26]. Therefore, other possible parameters including malnutrition, interference with maternal antibodies, changes in gut microbiota, and genetic susceptibility should be investigated [29,30].

## Conclusions

The licensed rotavirus vaccines showed lower efficacy in Bangladeshi trials and many investigator and policy makers are beginning to question whether the current vaccines may be useful in the country or whether an alternative vaccine will work better in this setting. In this context, our study provides important information on rotavirus genotypes that should be considered for the selection of vaccine strains, development of an alternative vaccine strategyand rotavirus vaccine introduction in national immunization programs.

## Abbreviations

icddr,b: International Centre for Diarrhoeal Disease Research, Bangladesh; RRC: Research Review Committee; ERC: Ethical Review Committee; ELISA: Enzyme-linked immunosorbent assay; EIA: Enzyme immuno assay; RT-PCR: Reverse transcriptase polymerase chain reaction; PAGE: Polyacrylamide gel electrophoresis dsRNA, Double-stranded ribonucleic acid; UV: Ultraviolet.

## Competing interests

The authors have no commercial disclosures to make with regard to this manuscript. No competing financial interests exist.

## Authors’ contribution

MHA carried out the data analysis, result interpretation, genotyping of rotavirus and preparation of the manuscript. ZH, SF, SM and SB helped in genotyping of rotavirus. ASGF initiated and designed the study. SKD and TA was involved in revising the manuscript critically for intellectual content. MR involved in data analysis, result interpretation and in revising the manuscript critically for intellectual content. All authors read and approved the final manuscript.

## Pre-publication history

The pre-publication history for this paper can be accessed here:

http://www.biomedcentral.com/1471-2334/13/320/prepub
